# Measuring Cochlear Duct Length – a historical analysis of methods and results

**DOI:** 10.1186/s40463-017-0194-2

**Published:** 2017-03-07

**Authors:** Robert W. Koch, Hanif M. Ladak, Mai Elfarnawany, Sumit K. Agrawal

**Affiliations:** 1 Biomedical Engineering, Western University, 1151 Richmond Street, London, ON N6A 3K7 Canada; 2 Department of Otolaryngology-Head and Neck Surgery, Western University, London, ON Canada; 3 Department of Medical Biophysics, Western University, London, ON Canada; 4 Department of Electrical and Computer Engineering, Western University, London, ON Canada; 5 London Health Science Centre, University Hospital, Room B1-333, 339 Windermere Rd, London, ON Canada

**Keywords:** Inner Ear, organ of Corti, Cochlear implants, Cochlear duct length, Computed tomography, Histology

## Abstract

**Background:**

Cochlear Duct Length (CDL) has been an important measure for the development and advancement of cochlear implants. Emerging literature has shown CDL can be used in preoperative settings to select the proper sized electrode and develop customized frequency maps. In order to improve post-operative outcomes, and develop new electrode technologies, methods of measuring CDL must be validated to allow usage in the clinic.

**Purpose:**

The purpose of this review is to assess the various techniques used to calculate CDL and provide the reader with enough information to make an informed decision on how to conduct future studies measuring the CDL.

**Results:**

The methods to measure CDL, the modality used to capture images, and the location of the measurement have all changed as technology evolved. With recent popularity and advancement in computed tomography (CT) imaging in place of histologic sections, measurements of CDL have been focused at the lateral wall (LW) instead of the organ of Corti (OC), due to the inability of CT to view intracochlear structures. After analyzing results from methods such as directly measuring CDL from histology, indirectly reconstructing the shape of the cochlea, and determining CDL based on spiral coefficients, it was determined the three dimensional (3D) reconstruction method is the most reliable method to measure CDL. 3D reconstruction provides excellent visualization of the cochlea and avoids errors evident in other methods. Due to the number of varying methods with varying accuracies, certain guidelines must be followed in the future to allow direct comparison of CDL values between studies.

**Conclusion:**

After summarizing and analyzing the interesting history of CDL measurements, the use of standardized guidelines and the importance of CDL for future cochlear implant developments is emphasized for future studies.

## Background

For years, the length and morphology of the human cochlea has been studied to determine the variations that exist between individuals and key features which may help predict a patient’s unique cochlear duct length (CDL). Since the creation of cochlear implants, there has been a greater interest in the dimensions of the cochlea and how it pertains to the insertion of the electrode. Considerable variations of CDL between individuals have been found [1–7] and the need to produce electrodes accordingly is becoming increasingly important to continuously improve postoperative outcomes.

With the advancement in electrode design and technology, it is becoming all the more important to understand CDL variation to identify trends and manufacture products accordingly. The introduction of variable length electrodes creates a need to develop a preoperative technique to determine the length of the patient’s cochlea, and to choose the correct electrode size for the patient [8, 9]. This selection of electrode is crucial as the electrode must provide proper cochlear coverage while avoiding any insertional trauma which may result from a deep insertion [9–15]. The lack of force feedback felt by the surgeon may result in inadvertent insertional trauma, therefore it is important to have a preoperative technique to select the appropriate electrode and depth of insertion [11]. Different lengths of electrodes are also needed depending on whether the specific electrode is made to be placed against the organ of Corti (OC) [16], mid-scala [17], or against the lateral wall (LW) and stimulate the peripheral processes and neurons [18].

Other techniques such as electric acoustic stimulation (EAS) require a reduction in insertional trauma during the implantation to preserve the residual hearing available in patients. This EAS technique will continue stimulating the high-frequency portions at the base of the cochlea with the cochlear implant while the low-frequency sections of the cochlea at the apex that are still functional are stimulated by standard acoustical stimulation [19, 20]. By minimizing the insertional trauma, more hair cells are left functional and an EAS technique can be used instead of a deep insertion, producing better postoperative success [21, 22]. A better understanding of the dimensions of the patient’s cochlea and intra-cochlear compartments will make minimizing the insertional trauma possible [23].

Frequency mapping of a patient’s cochlea is also an area of study which has become very important for postoperative success. As shown by numerous studies, the frequency distribution of the human cochlea has substantial variation between individuals due to the variation in cochlear length [7, 24, 25]. Studies have also found consistent frequency-to-length relationships [25–27], but in order to apply these equations, there must be reliable measures and methods of determining CDL and insertion depth on a specific patient [6]. Accurate measures of CDL and a technique to preoperatively determine this length are needed to maximize the effect of frequency mapping. Recent studies have used novel imaging techniques to create customized frequency maps and deactivate electrodes with overlapping stimulation patterns [15, 28–32].

Having proper methods of obtaining CDL and a reliable set of CDL values are important for the continuing development and improvement of cochlear implants. After reviewing the literature, there is no consistent definition of CDL or a consistent method to properly measure CDL. This topic has been covered by several studies, but many have differed in their approaches. The goal of this paper is to summarize the work being done to measure the CDL, and to give the reader the necessary tools to maintain the consistency that is needed for future studies to objectively compare lengths obtained across different cochleae.

## Methods of measuring CDL

**Fig. 1:**
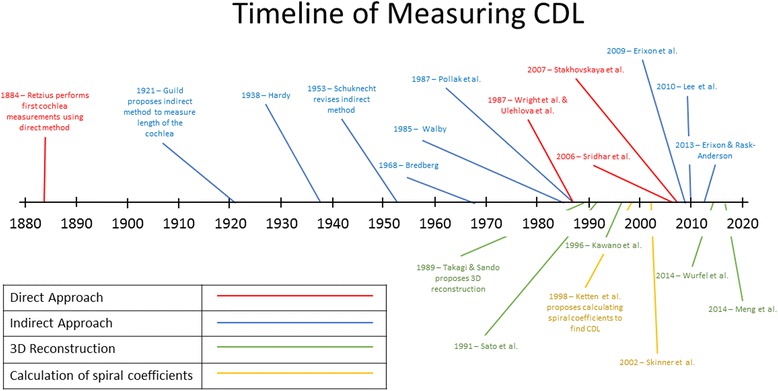
Timeline of studies involving measuring the entire CDL of the cochlea organized by method of measurement

The length of the cochlea has been evaluated by: a direct method; measuring the length from histologic sections by a micrometer under a microscope [4, 7, 33–36], an indirect method; graphically representing the cochlea by using landmarks from histologic sections or plastic casts [3, 4, 37–41], 3D reconstruction; reconstructing the cochlea by placing points on a cochlea and transferring the points to a computer as 3D coordinates [5, 24, 42–44], and by modeling the cochlea as a mathematical spiral function [6, 45–47]. As seen in the timeline in Fig. 1, the techniques have evolved with the technology that has been available. Each technique has been thoroughly explored, and the advantages and disadvantages of each are discussed below.

### The direct method

**Fig. 2:**
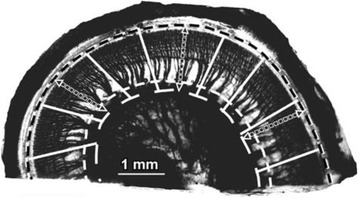
Direct method of measuring CDL. Length of the OC is measured along the dashed white and black line from a histologic section using a micrometer. Adapted with permission from Stakhovskaya et al., 2007

The direct method was the first method used to analyze the length of the cochlea [4]. This method uses histologic sections to directly measure the CDL with a micrometer. As seen in Fig. 2, the dashed black and white line is the line of the OC in which the measurements are taken at [35]. As stated by Hardy, there had been records for basilar membrane measurements in nine cochleae using this method before she published her landmark study in 1938 [4]. The lengths of the cochlea using this direct method were 33.5mm by Hensen in 1865, 28.0mm in one ear and 31.0mm in another by Waldeyer in 1873, and 33.0mm by Krause in 1876. In 1885, Retzius performed his study using five ears where he found the average length to be 33.5mm (as cited by Hardy, 1938) [4]. At this time, the average of 33.5mm was known to be the average length of the OC. Due to the small sample sizes in the previous studies, Retzius will be the first study used in this paper for a comparison, as seen in Table 1.

**Table 1:** Comparing CDL measurements made by multiple studies

Authors	Year	Location of CDL	Modality	Method	# of Samples	Mean (SD)	Range of Values
Retzius	1884	OC	Histology	Direct	5	33.5 (0.8)	32 – 34
Hardy	1938	OC	Histology	Indirect	68	31.52 (2.3)	25.26 – 35.45
Bredberg	1968	OC	Histology	Direct	35	34.0 (1.3)	30.3 – 37.6
Walby	1985	OC	Histology	Indirect	20	32.6 (2.1)	30.1 – 36.4
Ulehlova et al.	1987	OC	Histology	Direct	50	34.2 (2.9)	28.0 – 40.1
Pollak et al.	1987	OC	Histology	Indirect	9	28.4 (3.4)	24.0 – 33.5
Wright et al.	1987	OC	Histology	Direct	14	32.9 (2.6)	28.8 – 36.6
Takagi & Sando	1989	OC	Histology	3D reconstruction	1	36.4 (n/a)	-
Sato et al.	1991	OC	Histology	3D reconstruction	18	34.73 (2.9)	29.7 – 38.9
Kawano et al.	1996	OC	Histology	3D reconstruction	8	35.58 (1.4)	34.2 – 37.9
		LW	Histology	3D reconstruction	8	40.81 (2.0)	37.93 – 43.81
Ketten et al.	1998	OC^a^	In vivo CT	Spiral coefficients	20	33.01 (2.3)	29.07 – 37.45
Skinner et al.	2002	OC^a^	In vivo CT	Spiral coefficients	26	34.62 (1.2)	32.94 – 36.57
Sridhar et al.	2006	OC	Histology	Direct	7	33.31 (2.4)	30.5 – 36.87
Stakhovskaya et al.	2007	OC	Histology	Direct	9	33.13 (2.1)	30.5 – 36.87
Erixon et al.	2009	LW	Plastic casts	Indirect	58	42.0 (2.0)	38.6 – 45.6
Lee et al.	2010	OC	Histology	Indirect	27	30.8 (2.6)	25.5 – 35.1
Erixon & Rask-Anderson	2013	LW	Plastic casts	Indirect	51	41.2 (1.9)	37.6 – 44.9
Wurfel et al.	2014	LW	In vivo CBCT	3D reconstruction	436	37.9 (2.0)	30.8 – 43.2
Meng et al.	2016	LW	In vivo CT	3D reconstruction	310	35.8 (2.0)	30.7 – 42.2

^a^Measured LW and interpolated into the OC location

This method was used again in 1968 by Bredberg while studying the nerve supply to the OC. He found the mean length of 15 samples to be 34.0 mm [33]. Two more groups, Ulehlova et al. in 1987, and Wright et al. in 1987, used this method to calculate the length of the OC while investigating the hair cell distributions and the sensory cell density. Both of these groups used histologic sections under a microscope to estimate the length of the cochlea at the union of the pillar cells, which was now known to be the common location of measuring the OC [7, 37]. Ulehlova and Wright measured 14 and 50 samples with mean lengths of 32.9mm and 34.2mm, respectively [7, 34]. These results are summarized in Table 1.

Most recently, Sridhar et al. (2006) and Stakhovskaya et al. (2007) published studies attempting to create a frequency-position function for the spiral ganglion cells in the cochlea using Greenwoods previous work [35, 36]. Using this direct approach, they measured the length of the OC on 9 human cadaveric samples finding an average length of 33.13mm.

In general, the direct method was used while studying the spiral ganglion cells or the nerve supply to the cochlea. A specific view clearly showing the nerves (as seen in Fig. 2) is an excellent view to be able to directly measure the length of the OC by this method.

### The indirect method

**Fig. 3:**
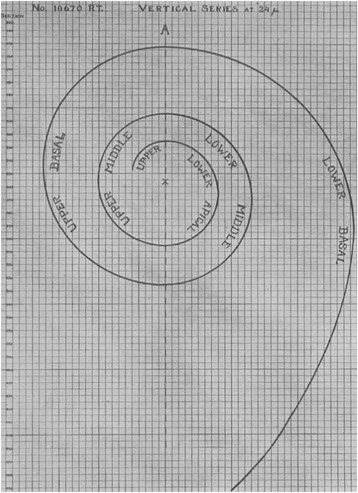
Indirect method of 2D graphical reconstruction. By taking histologic slices parallel to the cochlear axis and projecting points from the histologic sections down onto a template (as seen above), the CDL was calculated. Adapted with permission from Hardy., 1938

In 1921, Guild strove to develop a consistent protocol to analyze the cochlear structure after studying the result of detonation injuries on the human ear from World War One [37]. Using 2D graphical reconstructions of histologic sections of the cochlea (as seen in Fig. 3), she proposed to evaluate the approximate lengths of all portions of the OC when cut in serial sections. Relevant landmarks were marked on the sections and the locations were projected onto a 2D plane. This technique required good fixation and embedding of the cochlea, and serial sections of the cochlea in the plane of the modiolar axis. In addition, Guild was the first to propose measuring the length of the OC at the center of the union of the pillar cells. With future studies using this universal method, the lengths obtained by different cochleae could be directly compared. This method was further improved by Schuknecht in 1953 to attempt to account for the 3D shape of the cochlea [48].

Hardy (1938) was the first to employ this indirect method of graphic reconstruction to measure the length of the OC in 68 samples [4]. Having an average length of 31.52mm with a variation of roughly 10mm, it was similar to the previous work done by the direct method. Hardy presented the first major study involving a large population of cochleae aimed at finding the length of the human cochlea.

Following Hardy, two groups used Schuknecht’s revisions to measure the OC. Walby in 1985 measured 20 samples to have an average length of 32.6mm and Pollak et al. in 1987 measured 9 samples with a mean of 28.4mm, also summarized in Table 1 [38, 39].

The indirect method was not used for some time after Pollak et al.’s study as computers had begun to be utilized for 3D reconstruction, as will be discussed later. The indirect method was used again by Erixon et al. in 2009 when they began analyzing variations in the anatomy of the cochlea and how it affected cochlear implants [3]. Using plastic casts created by the method explained by Willbrand et al. [49] and Wadin [50], the length of the LW was measured as the OC was not visible. The results from these measurements for the 73 samples varied from 38.6mm to 45.6mm with a mean of 42.0mm. This study still used the indirect approach, but instead of preparing serial histologic sections, consistent photographs were taken from above the plastic casted cochleae, and the 2D reconstruction was done from the images. Erixon performed another study on different plastic casts in 2013 finding very similar results as the previous study [41]. The 51 samples used had a mean length of the LW of 41.2mm and a range of 37.6mm to 44.9mm.

Most recently, Lee et al. in 2010 used Guild’s and Schuknecht’s method to analyze the postoperative performance of cochlear implants using histologic sections [51]. During this study, they found the mean CDL at the OC of 27 samples to be 30.8mm.

### 3D reconstruction

As of 1989, the only two methods used to analyze the length of the cochlea were the direct and indirect method. Having the tools to create a new 3D reconstruction of the cochlea, Takagi & Sando explored the accuracy of this new technique by comparing new measurements from the 3D reconstruction to measurements from the direct and indirect method on the same cochlear sample [24]. Takagi & Sando performed their measurements by plugging in coordinates based on histologic sections into a computer program and reconstructing the 3D structure to find the length of the cochlea. Comparing the 3D reconstruction method to the indirect method (developed by Guild and improved by Schuknecht), it was clear that the cutting angle of the sample had a large effect on the CDL. In brief, the cutting angle effect is the effect of not choosing the correct plane for 2D graphical reconstruction, which misrepresents the cochlear dimensions, as will be discussed later.

After Takagi & Sando highlighted the challenge of using the indirect approach, Green et al. (1990) furthered their hypothesis that 3D reconstruction has several advantages over 2D reconstruction while studying the temporal bone [52]. In 1991, Sato et al. utilized this 3D approach to measure CDL. After measuring the length of the OC of 18 samples (repeated 3 times on each sample), they found a mean of 34.73mm. It is important to note that Sato et al. measured the length of the outer and inner margins of the basilar membrane and took the mean to predict the location of the OC.

**Fig. 4:**
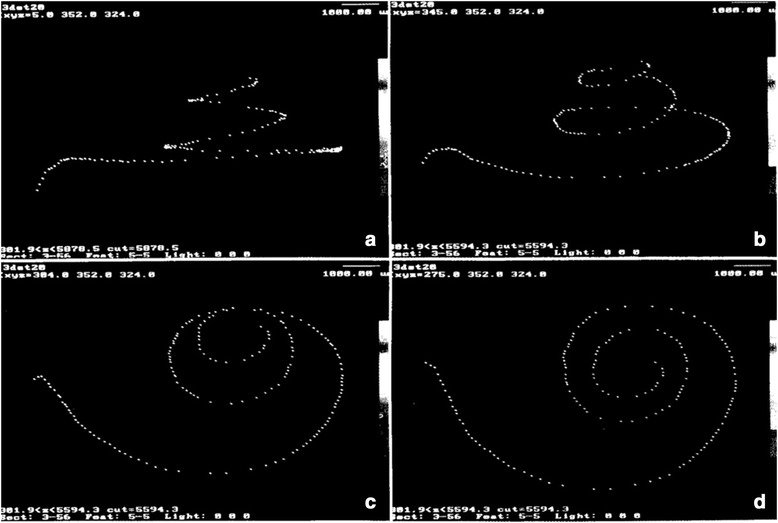
Method of 3D reconstruction first explored by Takagi for the human cochlea. Points in figures correspond to points placed on a histologic section, and recreated into a 3D shape using a computer, as shown by the varying views in panels **a**, **b**, **c**, and **d**. Adapted with permission from Kawano et al., 1996

In 1996, Kawano et al. was the next group to analyze the length of the cochlea by means of the 3D reconstruction technique (Fig. 4), and compared these to the previous 2D techniques [5]. They also measured the length of the LW, inner wall, and Rosenthal’s canal. His study furthered the hypothesis that using the 3D reconstruction produces different (possibly more accurate) results than the 2D method described by Guild and Schuknecht, finding a mean percentage difference based on cutting angle of 7.55%. The final average measurements at the OC, LW, inner wall and Rosenthal’s canal were 35.58mm, 40.81mm, 18.29mm and 15.98mm, respectively. This study promoted the prediction that using the traditional 2D indirect approach was underestimating the length of the OC, likely due to the cutting angle effect, as the previous two studies by Takagi & Sando and Sato et al. also predicted [24, 42].

Wurfel et al. in 2014 measured the cochlear length using cone beam computed tomography (CBCT) scans from 436 patients using a 3D planar reconstruction [43]. When the 3D reconstruction technique was used with high resolution CT scans, the time required to perform the reconstruction was reduced significantly compared to when histologic sections were used. CDL was only measured at the LW as the OC was not visible due to the lack of resolution associated with clinical CT technology. After analyzing 436 clinical scans, they found lengths of the LW of 30.8 – 43.2 mm (the largest range of all studies) with an average length of 37.9mm.

Most recently in 2016, Meng et al. measured CDL at the LW while following the procedure developed for clinical CT scans by Wurfel et al. [44]. After analyzing 310 of their own clinical CT scans, they found a range of 30.7mm to 42.2mm with an average of 35.8mm.

### Calculation of spiral coefficients

By 1998, CT imaging had proven to be useful to identify malformations of the cochlea prior to surgery or to assess the final location of electrode after surgery [53–56]. Looking to utilize pre and post-operative CT scans, Ketten et al. were interested in finding the insertion depths of the Nucleus cochlear implant arrays as well as calculating the CDL based on in vivo CT scans [6]. Because their focus was pertaining to the location of an electrode array, they measured the canal length in the centroid of the fluid filled space which was considered comparable to the OC. Instead of past techniques that were used, they used a technique that approximates the cochlear structure based on spiral coefficients that had been used with mammalian species before, but not on humans [57]. By calculating different dimensions of the cochlea, (apical diameter and spiral constant) they were able to approximate the cochlea with an Archimedean spiral equation, with the assumption that the length of the cochlear spiral length differs between patients but the general shape of the curve does not. The results from this study agreed with all the previous studies finding a mean length of 33.01mm with 20 patients.

A group led by Skinner imaged an additional 13 patients and added them to the original 13 patients from Ketten’s study [47]. They decided that the cochlea resembles the Archimedean spiral for the entire length except the hook region at the base of the cochlea. In order to account for this, they added an extra component representing the hook region, creating a mean length of 34.62mm for the centroid of the cochlea.

**Fig. 5:**
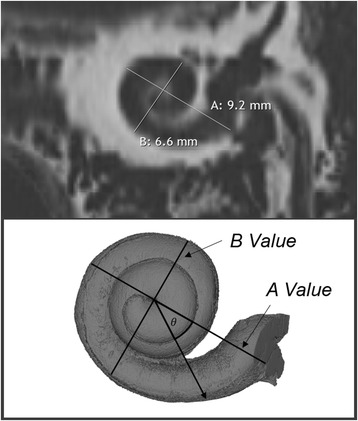
By measuring spiral coefficients such as the ones above, the CDL can be calculated using typical spiral equations. A value is defined as the largest distance from the round window to the contralateral wall, B value as the distance perpendicular to ‘A’ and $\theta$ as the insertion angle

Escude et al. in 2006 improved upon Ketten’s work by proposing a formula that requires only one spiral coefficient to find approximate lengths of different turns of the cochlea [46]. Using CT scan data from 42 patients, they predicted where the electrode may sit using a single measurement by a surgeon. This measurement known as the ‘A’ value is the largest distance from the round window to the opposing LW of the cochlea (as seen in Fig. 5). In this study, it was found that measuring the value ‘A’ can help predict the insertion depth of the electrode and the length of the LW at a particular insertion angle using a basic spiral function. Despite not measuring the entire cochlear length, this was the first step to having a pre-operative technique to allow surgeons to pick the correct size electrode for the specific patient.

Building on Escude et al.’s work to create a way to measure the LW based on simple linear measurements, Alexiades et al. in 2012 found the relation between the measured A value and CDL at the OC [45]. Using Escude et al.’s spiral equation relating the ‘A’ value to Basal Turn Length (BTL) and using Hardy’s data to plot a regression line relating BTL vs CDL, a linear equation was developed to approximate the length of the cochlea at the OC. This length was also measured at the electrode location, accounting for the average radius of a Med-El electrode. Future studies must work to validate this equation and assess the accuracy of this equation.

## Discussion

### Measurement method

Table 2 presents a summary of CDL values obtained from all studies explicitly reporting CDL values categorized based on the method of measurement. First, the indirect method has been reported to underestimate the cochlear length at the OC [5, 22, 26, 35]. The original method by Guild and used by Hardy ignored the height of the cochlea as a factor and did not include the unique shape of the hook region [26]. According to Bredberg, these errors will result in an error of approximately 1.0 – 1.5 mm. Comparing Hardy’s results to the other studies (as seen in Table 1) shows this underestimation of the CDL at the OC. Schuknect’s revisions in 1953 accounted for some of these errors, but still had the cutting angle effect which could give varied results while measuring the same cochlea. The cutting angle effect is the error associated with projecting histologic slices that are not in line with the modiolar axis, and rather are at a certain unknown angle causing a false representation of the diameter of the cochlea, as seen in Fig. 6. As explored by Kawano et al., slicing the cochlea by a plane at an angle of 5° to 29° away from the modiolar axis will produce errors from 4 to 13%, respectively. In Takagi & Sando’s example, the CDL measured with the 3D reconstruction was 36.3mm while the indirect approach used by previous researchers measured the CDL to be 30.8mm giving a 15.4% difference [22]. Even though Takagi & Sando only used one sample in their study, it effectively demonstrated how the cutting angle affects the reliability of the 2D reconstruction method. However, for LW, the indirect method overestimated CDL compared to other methods. This is likely due to the modality, and the use of plastic casts by Erixon et al. (as will be discussed later).

**Table 2:** Comparing Methods used to Calculate CDL in Various Studies

Method	Years	Where CDL is Being Measured		Range of Values		Mean Length		# of Samples	
		OC	LW	OC	LW	OC	LW	OC	LW
Direct	1884 – 2007	6	0	28.0 – 40.1	-	33.79	-	100	
Indirect	1921 – 2013	4	2	24.0 – 36.4	37.6 – 45.6	31.31	41.6	124	109
3D reconstruction	1989 – 2014	3	3	29.7 – 38.9	30.7 – 43.2	35.04	37.07	27	754
Spiral Coefficients	1998 – 2016	2^a^	1	29.07 – 37.45	30.76 – 37.41	33.92	37.41	46	148

^a^Calculated at centroid of fluid filled space in CT image to represent OC measurements

Means calculated by multiplying mean length by the number of samples, summating this value from each study using the selected method, and dividing by total number of samples analyzed with this method

Second, the direct method was reported to be more accurate than the indirect method since it is not susceptible to the cutting angle effect [5]. It is however vulnerable to the viewing angle effect; the angle at which the histologic section is viewed underneath the microscope. After investigation by Kawano et al., it was concluded that the viewing angle was less influential to the total length of the cochlea than the cutting angle effect [5]. The length of the cochlea found by this direct method were on average longer than the indirect method, and closer to the measurements found by the 3D reconstruction method.

Third, the 3D reconstruction is the most accurate method to measure the length of the cochlea [5, 22, 35, 36, 45]. These reconstructions consider the entire complex 3D shape of the cochlea and are not susceptible to the cutting or viewing angle effects [5]. By plotting points on histologic sections or on CT scan data, this information can be inserted into a 3D coordinate system to create a 3D model.

Lastly, the spiral coefficients method was created to find a more efficient way to model the cochlea while staying as accurate as possible. It was known that 3D reconstruction was the most accurate method but the time involved to perform this reconstruction with histologic sections, as previously described, made it a difficult method to use [24]. It was not until Wurfel et al. in 2014 that the resolution and technology available in a CT scan was used to accurately represent the cochlea in a 3D model. Instead, calculating a few dimensions of the individual cochlea from CT scans allows a general mathematical spiral to be fitted according to the uniqueness of the cochlea being analyzed. This approximation of the spiral of the cochlea is far less time consuming than 3D reconstruction while maintaining its accuracy. The data from the spiral coefficients method is on average the closest to the 3D reconstruction data, meaning it may be the second best representation of the cochlea, next to the 3D reconstruction. With the introduction of variable lengths of electrodes, the spiral coefficient methods allow a surgeon to approximate the length of the patient’s cochlea using a single linear measurement on preoperative scans.

### Data acquisition modality

The modality used to analyze the length of the cochlea has also changed considerably. Before 1998, the only modality used to measure the CDL was histologic sections. The OC and basilar membrane is easily discernable so placing points or taking measurements is not a difficult task while measuring the CDL. Unfortunately, there are challenges associated with histologic sections, including increased time and tissue shrinkage [58–61].

In 1998, Ketten and Skinner used clinical CT scans to visualize the cochlea. Their attempt to calculate the spiral coefficients using this data has been expanded on in later studies [45, 46] and has proved to successfully approximate the length of the cochlea. In 2014, Wurfel et al. utilized higher resolution CBCT scan data to reconstruct the cochlea and provide a new set of measurements, as creating 3D models from CT scan data is very quick compared to creating models from histologic sections [43]. Despite the advantages of CT, the resolution is limited by the technology available and the level of safe radiation dosage. Even if cadaveric samples are used for high resolution CT scans, histology is far more accurate and has the ability to discern the inner structures of the cochlea.

The last modality used was plastic casts by Erixon et al. in 2009 and 2013. They extracted the cochlea from cadaveric specimens and stained and hardened them into plastic casts [3, 41]. Erixon et al. then used an indirect approach to measure the length of the LW. By capturing an optical image at a consistent distance above the cochlea, their method may still cause measurement errors if the acquisition angle is not kept consistent or the orientation of the cochlea is different across different samples.

### Location of measurements

Depending on the study and the modality used to measure the CDL, the location of the measurement varied. Every study in which histologic sections were used, the measurement was taken at the OC due to the ease of discerning internal structures.

Studies using CT scan data primarily measured the CDL at the LW. In clinical CT images, the internal structures of the cochlea are not visible, therefore the only reference point which can be consistently seen to measure the CDL in these images is the LW. Ketten et al. and Skinner et al. attempted to overcome this challenge by interpolating into the centroid of the fluid filled space to an approximate location of the OC and thus, maintaining consistency with previous studies [6, 47].

While the OC measurement is taken directly at the union of the pillar cells, there is no consistent definition of where the LW measurement should occur. Depending on the study and the modality used, the LW may be measured at different locations. While using plastic casts and clinical CT, the LW was considered to be the most lateral part of the cochlea, due to the inability to view any internal structures [3, 41, 43, 44]. With micro-CT, partial views of the internal structures are possible so CDL measurements may occur at an extension of where the basilar membrane would meet the LW. In histology, the LW was considered to be the LW of the scala tympani instead of the most lateral location of the entire cochlea [5]. These inconsistencies will be responsible for some of the variation existing within the LW measurements.

It is also important to note that the selection of these points on the LW and OC are subject to inter-observer variability, as different people performing the measurement will choose different locations on the same cochlea. This is especially true with studies utilizing CT scans, as the basilar membrane and OC are not visible.

### Electrode position

In 2012, Alexiades et al. proposed to measure the CDL at a new location to simulate where a LW electrode may be placed. By finding a location in between the LW and the OC and accounting for the radius of a typical standard commercial electrode, they developed an equation to find the length of the cochlea at this electrode location. There are no studies yet verifying different lengths of CDL at this location.

### Measurement starting point

In each of the presented studies, the CDL was measured using a unique protocol. One aspect that varied among them was the location in which the measurement began, or the *zero reference angle*. While some groups have started at the middle of the round window, others start at the inferior edge of the round window [1, 2]. In 2010, there was a consensus panel held which defined the zero reference angle as the center of the round window [62]. Another related result was a consistent 3D cylindrical coordinate system of the cochlea with the z-axis through the modiolus.

### Measurement ending point

Another issue noted by Erixon et al. is identifying the ending point for measuring the length of the cochlea [3]. Most studies claimed to end their measurements at the apex, however the definition of the apex varied. Studies based on histologic sections defined the apex as the end of the OC, however the OC does not anatomically extend to the cochlear apex. In contrast, studies measuring the length at the LW did not define how the cochlear apex was determined. According to Erixon & Rask- Anderson, two authors measuring the same cochlear models produced a difference in CDL of approximately 1mm due to their subjective differences in the selection of the apex [41]. In addition, clinical CT scans may not have the sufficient resolution to accurately identify the apex, leading to error while using this modality.

Instead of attempting to identify the apex, other studies have concluded their measurements after the second turn to find the two turn length (2TL) [63]. There is interest in finding the 2TL in addition to the CDL to try to mitigate some of the variation that exists between cochleae. As stated by past studies, the majority of the variation of the cochlea exists within the apical turn of the cochlea [4, 41, 45]. By calculating the 2TL (basal and middle turn only) instead of the CDL, less variation is present and linear relationships such as Alexiades et al.’s relationship between ‘A’ and cochlear lengths become valid. Alexiades et al. and Erixon & Anderson have both shown better correlation with 2TL as opposed to complete CDL [41, 45]. While 2TL may be sufficient to select electrode length, the entire CDL is essential to use the Greenwood Equation for frequency mapping.

### Current challenges with measurement of CDL

While the advancement of technology and the methods used to measure CDL has improved the accuracy of new measurements, it has also led to major inconsistencies with past studies. As new studies are performed, certain protocols and guidelines such as those agreed upon in 2010 (where to start the measurement) [62] must be followed to allow for comparisons between all CDL studies. For future studies, the most accurate methods and modalities should be used to gather new, impactful data. As stated above, the 3D reconstruction method has been proven to provide the most accurate CDL measurements, while the best modality depends on the situation. Although high resolution micro-CT imaging and histology offers visualization of the internal structures, the ability to image large numbers of patients in a clinical setting with clinical CT is extremely valuable. Following standardized methods such as where to take measurements at the entrance, middle and apex of the cochlea is also becoming critical in order to objectively compare past studies, and allow for reliable calculations.

## Conclusion

CDL measurements have had a very interesting history and from 1884 to the present day, there have been a large number of studies explicitly measuring the entire length of the cochlea. The importance of CDL measures are becoming increasingly important as current technologies improve, and frequency maps based on the patient’s individual cochlear anatomy are developed. Standardization of measuring CDL and related lengths will help improve accuracy and consistency of calculations and will lead to further advancements in the field of cochlear implants.

## Abbreviations

2TL: Two-turn length
BTL: Basal turn length
CBCT: Cone beam computed tomography
CDL: Cochlear duct length
CT: Computed tomography
EAS: Electric acoustic stimulation
LW: Lateral wall
OC: Organ of Corti
